# Extrachromosomal circular DNA as a novel biomarker for the progression of colorectal cancer

**DOI:** 10.1186/s10020-025-01164-y

**Published:** 2025-04-01

**Authors:** Quanpeng Qiu, Yi Ding, Xiaolong Guo, Jing Han, Jiaqi Zhang, Yaping Liu, Junjun She, Yinnan Chen

**Affiliations:** 1 Department of General Surgery, The First Affiliated Hospital of Xi’an Jiaotong University, Xi’an, Shaanxi China; 2 Center for Gut Microbiome Research, Med-X Institute, the First Affiliated Hospital of Xi’an Jiao Tong University, Xi’an, Shaanxi China; 3 Department of High Talent, The First Affiliated Hospital of Xi’an Jiaotong University, Xi’an, Shaanxi China; 4 Department of Gastroenterology, The First Affiliated Hospital of Xi’an Jiaotong University, Xi’an, Shaanxi China; 5 Hubei Province Key Laboratory of Precision Radiation Oncology, Wuhan, China

**Keywords:** Colorectal cancer, Extrachromosomal circular DNA, Biomarkers

## Abstract

**Background:**

Extrachromosomal circular DNA (eccDNA) has potential in tumor diagnosis, particularly for improving diagnostic accuracy and early cancer detection; however, many challenges remain in its application to clinical practice.

**Methods:**

We conducted a Circle‐Seq analysis on clinical samples at different stages of colorectal cancer progression to examine the dynamic changes of eccDNA during the progression of colorectal cancer. We used breakpoint-specific PCR to verify candidate eccDNAs identified by Circle‐Seq. The results were further validated using the AOM/DSS-induced colorectal cancer model.

**Results:**

There was an increase in the abundance of eccDNA with the progression of colorectal cancer. The genes associated with these eccDNA molecules were primarily related to signaling pathways involved in tumor development and metastasis. Our analysis also revealed that eccDNA abundance positively correlates with gene expression, and eccDNA derived from specific genes has potential value for the early diagnosis of tumors.

**Conclusions:**

This study revealed a connection between eccDNA and colorectal cancer progression and highlights the clinical potential of eccDNA for the early diagnosis of colorectal cancer.

**Supplementary Information:**

The online version contains supplementary material available at 10.1186/s10020-025-01164-y.

## Introduction

Colorectal cancer (CRC) is one of the most common and lethal malignant tumors worldwide. It has a high incidence and mortality rate and has shown an increasing trend in recent years (Siegel et al. 2023). More than 90% of colorectal cancers originate from adenomas (Siegel et al. 2023; Dekker et al. 2019). Polyps with a certain malignant potential, such as adenomatous polyps, may develop into adenomas. In these cases, cells begin to show dysplasia, which is a form of precancerous lesion (Chen et al. 2021; Becker et al. 2022).

Therefore, the early detection and diagnosis of colorectal cancer is essential to improve treatment outcomes and survival rates. As the disease progresses, changes in the intestines may be directly observed by colonoscopy (Dekker et al. 2019; Brenner et al. 2024). Methods such as imaging and tumor marker detection may also be used for further diagnosis (Dekker et al. 2019; Patel and Dominitz 2024). In addition, with the development of liquid biopsy technology, the analysis of circulating tumor DNA (ctDNA) in the blood has become an important method to monitor changes in colorectal cancer (Malla et al. 2022). There are limitations to early detection techniques for colorectal cancer, such as colonoscopy, which is effective but invasive, whereas the sensitivity and specificity of the fecal occult blood test are not perfect (Dekker et al. 2019; Patel and Dominitz 2024; Shaukat and Levin 2022). Furthermore, ctDNA liquid biopsy is limited by the low capture of ctDNA from plasma. Other markers obtained by screening also have low specificity and sensitivity and an inability to determine the tissue source of the tumor (Malla et al. 2022; Ignatiadis et al. 2021). Therefore, it is necessary to identify novel biomarkers for the early diagnosis of CRC.

Extrachromosomal circular DNA (eccDNA) is circular double-stranded DNA formed by shedding from chromosomes. Its potential use in oncology has been gradually realized since its discovery (Turner et al. 2017; Wu, et al. 2019; Hung et al. 2021). Extrachromosomal circular DNA is generally divided into two main categories, extrachromosomal circular DNA (eccDNA) and extrachromosomal DNA (ecDNA). EcDNA has been found in a relatively wide range of sizes, from hundreds of thousands to millions of base pairs. In tumor cells, larger ecDNA may be more conducive to carrying multiple genes, particularly oncogenes and their regulatory elements, thereby promoting the proliferation and survival of tumor cells. EccDNA generally refers to relatively small pieces of circular DNA, which may range from tens to thousands of base pairs. They are involved in cell-to-cell communication as signaling molecules or play a role in fine-tuning gene expression. Currently, eccDNA has been found in a variety of tumor types and is present during the early stages of cancer (Yang et al. 2022). This suggests that it may have broad applicability as a marker for early tumor diagnosis. Additionally, changes in the level of eccDNA may correlate with the growth and spread of tumors (Verhaak et al. 2019). Therefore, it may be used as an indicator of disease progression, thereby improving the success of treatment. Certain eccDNAs may be associated with the tumor response to certain treatments (Nathanson et al. 2014; Yan et al. 2020; Leen et al. 2022), which may aid in the development of personalized treatment options.

Compared with other tumor markers, eccDNA has significant advantages. It is abnormal during the early stages of tumorigenesis. When normal cells begin to transform into tumor cells, along with genetic instability, eccDNA forms and accumulates oncogene fragments. If sensitive tests can be developed to detect eccDNA in blood or tissues, it may be possible to detect tumors at an early stage, even before clinical symptoms are evident. Because of its strong association with disease progression in certain cancers, it may exhibit higher specificity as a tumor marker (Verhaak et al. 2019; Kim et al. 2020). In terms of sensitivity, the circular structure of eccDNA imparts greater stability, which makes it easier to detect and analyze (Wu et al. 2019). In addition, eccDNA may be present in body fluids at an early stage of cancer, making it a potential marker for early detection (Sin et al. 2020; Sin et al. 2021; Luo et al. 2023). In this study, we analyzed the evolution of eccDNA during the occurrence and development of CRC and its potential as an early diagnostic marker for CRC. The results provide an important foundation for further studies of eccDNA as a diagnostic tumor marker.

## Materials and methods

### Case recruitment

All human colorectal polyp (n = 17) and adenoma (n = 14) tissues, tumor tissues (n = 29) and adjacent normal tissues (n = 5) from CRC patients and intestinal epithelial tissues from healthy individuals (n = 5) were collected from the First Affiliated Hospital of Xi’an Jiaotong University (Xi’an, China). The diagnosis of CRC, colorectal polyp and adenoma was jointly determined by two professors of pathology. This study was reviewed and approved by the Medical Ethics Committee of the First Affiliated Hospital of Xi’an Jiaotong University.

### EccDNA extraction and enrichment

DNA was extracted using the DNeasy Blood and Tissue kit (Qiagen) according to a standard protocol for high-quality extraction, following the manufacturer’s instructions. DNA concentration was determined using a NanoDrop spectrophotometer. Then FastDigest MssI (Thermo Scientific) and Plasmid-Safe ATP-dependent DNase (Epicenter, Madison, WI, USA) were used to remove mitochondrial circular DNA and the residual linear DNA. The purified samples were used as templates to amplify eceDNA using φ29 polymerase amplification (REPLI-g Midi Kit, QIAGEN, Germany). The amplification reactions were conducted at 30 °C for 16 h and library preparation was performed using the NEBNext® Ultra II DNA Library Prep Kit for Illumina (New England Biolabs). The sequencing was performed on an Illumina NovaSeq 6000 sequencer with 150 bp paired-end mode following the manufacturer’s instructions.

### Circle-sequencing analysis

Raw reads were filtered to remove the adaptors and low-quality bases using fastp (Chen 2023), and were mapped to the human genome reference (GRCh38) using BWA-MEM (Li and Durbin 2009). Then, we used Circle‐Map (Prada-Luengo et al. 2019) to detect the circular DNA, which was further annotated by bedtools (Quinlan and Hall 2010). The Circular DNA less than 10 kb in length was considered as EccDNA for downstream analysis.

EPM is the number of EccDNA per million mapped reads and is normalized according to the sequencing depth of each sample. EPM can be further normalized using the length of the genomic elements or chromosome as needed. The “eccDNA abundance” of each gene was calculated based on the number of eccDNAs per million bases of gene length.

### Breakpoint-specific PCR

EccDNA validation was performed by outward PCR, using original eccDNA as the template. Primers were designed according to the breakpoint site, and the resulting PCR product was loaded on the 1% agarose gel for electrophoresis.

### RNA extraction and RNA-seq analysis

Total RNA was extracted from tissue specimens using Trizol reagent (Solarbio, Beijing, China) according to a standard protocol. RNA-seq data was filtered to remove the adaptors and low-quality bases using fastp. Then Salmon and tximeta were used for gene-level quantification, and the DESeq2 R package was used for the detection of differentially expressed genes with log2 transformed fold-change > 1 and Benjamini–Hochberg adjusted p-values < 0.001(Love et al. 2020; Love et al. 2014). TPM is counts per length of transcript (kb) per million reads mapped. The spearman’s correlation between gene expression and eccDNA abundance was calculated using the cor-test function of the R package rstatix. The Metascape was used to perform the enrichment analysis of pathways and biological processes with eccDNA-related genes.

### AOM/DSS-induced colorectal cancer model

Nine-week-old male C57BL/6 mice were first intraperitoneally injected with a single dose of 10 mg/kg AOM (azoxymethane, Aladdin, Shanghai, China), followed by three cycles of 1.5% DSS (cycle 1), 1.8% DSS (cycle 2 and cycle 3) (dextran sulfate sodium, MP Biomedicals, Solon, Ohio, USA) administration to induce colorectal tumors. At the end of the experiment, the mice were euthanized. All animal experiments were approved by the Institutional Animal Care and Use Committee of Xi’an Jiaotong University.

### Statistical analysis and reproducibility

All data were presented as the means ± standard deviation, and statistical analyses were performed by the GraphPad Prism (version 8, San Diego, CA, USA) with Student’s T-test. For all analyses, *p* < 0.05 was considered statistically significant. Representative images of micrographs were shown to represent reproducible data from various experiments using micrographs.

## Results

### Genome‐wide distribution of eccDNA correlates with the progression of colorectal cancer

**Fig. 1:**
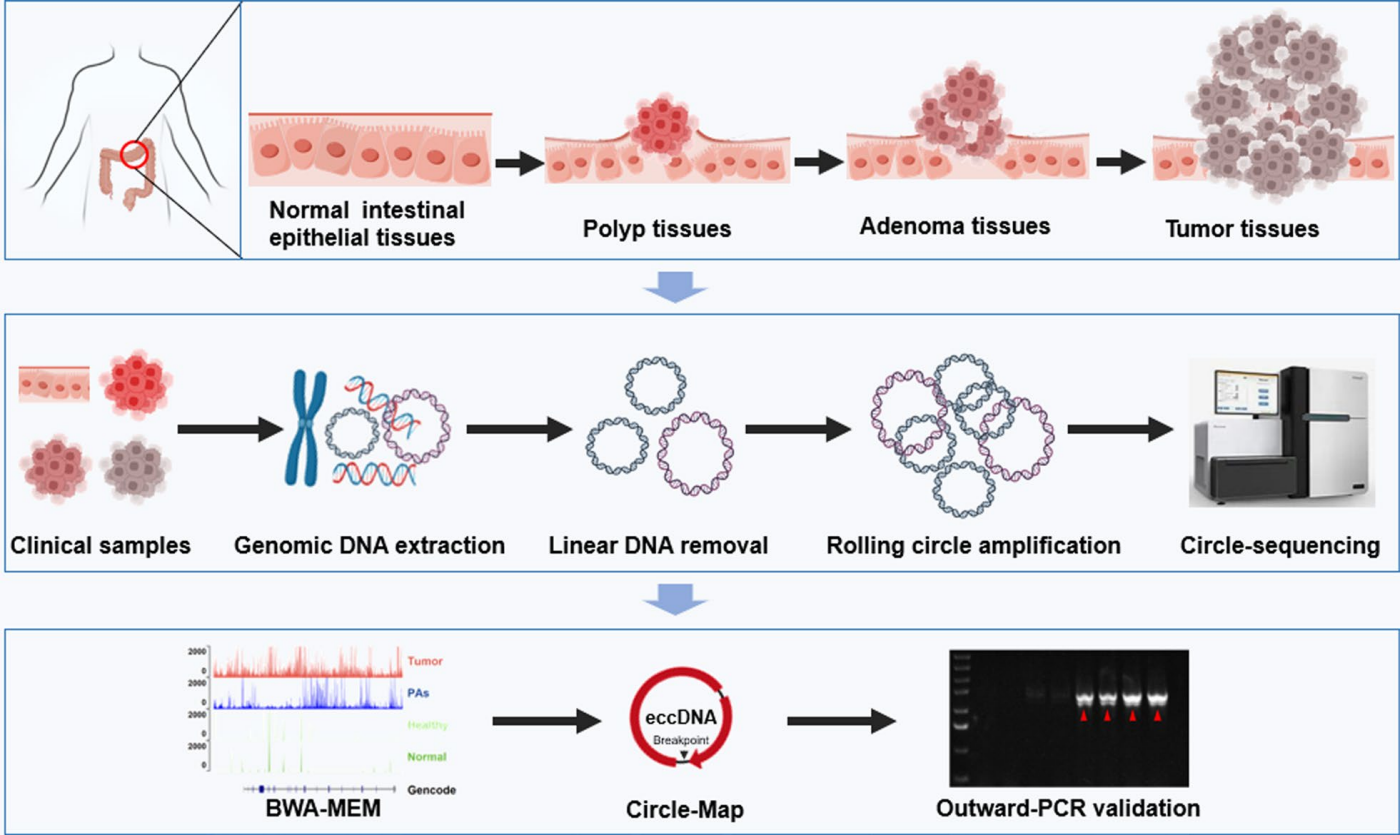
Schematic diagram of the experimental workflow. Patients with colorectal polyps (n = 17), adenomas (n = 14), and tumors (n = 29) and healthy individuals (n = 5) were recruited for this study. Diseased tissues from patients and normal intestinal epithelial tissues from healthy individuals were collected by colonoscopy. Next, whole-genome DNA including linear and circular DNA, was extracted from the clinical samples. The linear DNA was then removed by exonuclease and the purified circular DNA was amplified through rolling circle amplification. Raw reads were mapped to the human genome reference (GRCh38) using BWA-MEM, Circle‐Map was applied to identify eccDNA from sequencing data. Finally, the circular structure of eccDNA was verified by outward PCR

**Fig. 2:**
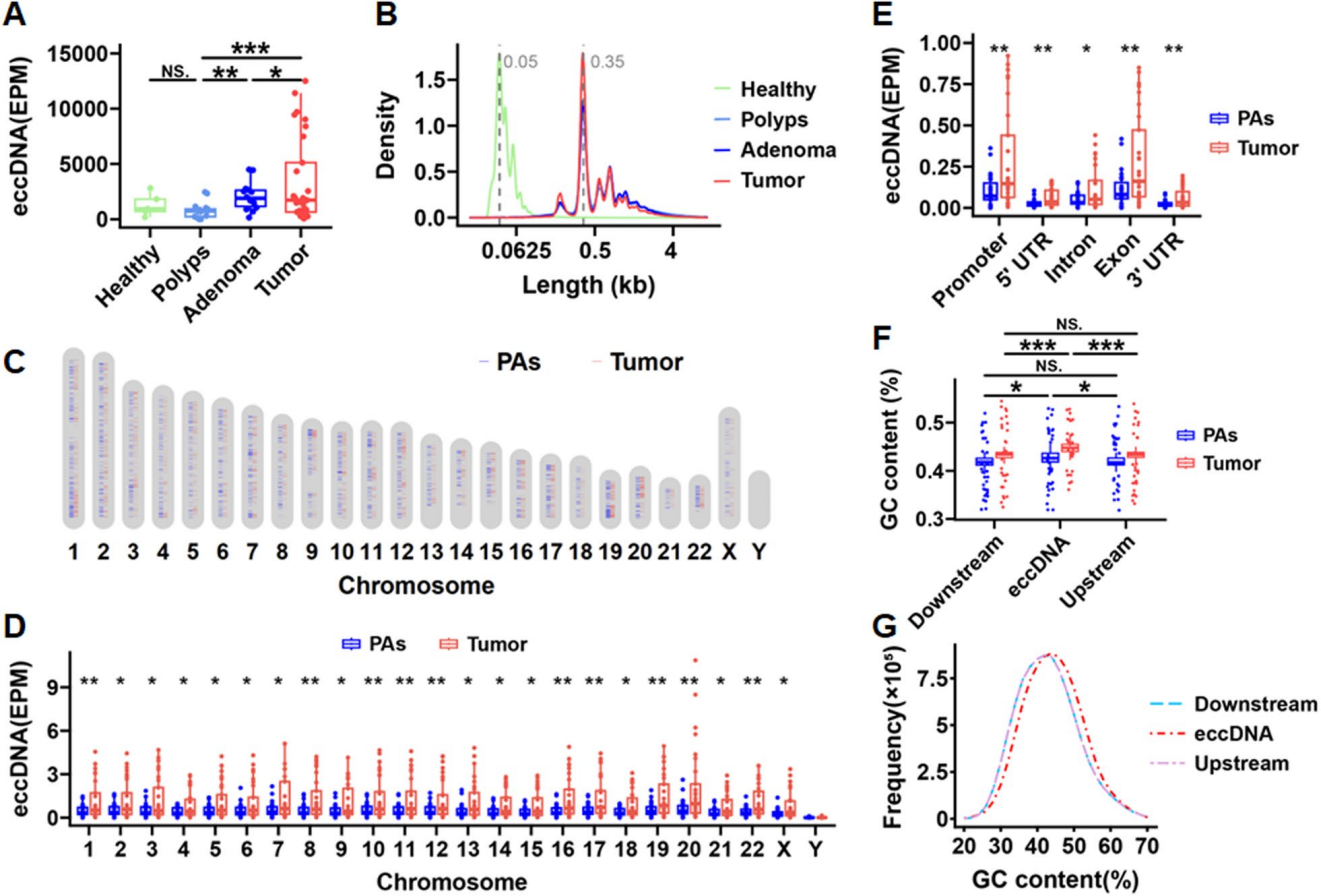
Characteristics of eccDNA at various stages of CRC occurrence and development. **A** EccDNA abundance after removal of linear DNA in intestinal epithelial tissues from healthy individuals (Healthy), colorectal polyp (Polyp) and adenoma tissues (Adenoma), as well as tumor tissues from CRC patients (Tumor). **B** Density distributions of eccDNA in intestinal epithelial tissues from healthy individuals, colorectal polyp and adenoma tissues, as well as tumor tissues from CRC patients. **C** Overall chromosomal distribution of eccDNA across the genome in colorectal polyp and adenoma tissues (PAs) and tumor tissues (Tumor). **D** Differences in chromosomal origin of eccDNA in colorectal polyp and adenoma tissues (PAs) and tumor tissues (Tumor). **E** Genomic elements distribution of eccDNA in colorectal polyp and adenoma tissues (PAs) and tumor tissues (Tumor). **F** Differences in GC content of eccDNA and its downstream and upstream regions of equivalent length in colorectal polyp and adenoma tissues (PAs) and tumor tissues (Tumor). **G** GC content distribution of eccDNAs and its downstream and upstream regions of equivalent length. Data were statistically analyzed using a Student t test. *p < 0.05, **p < 0.01, ***p < 0.001

We first assessed eccDNA levels in colorectal polyp (n = 17), adenoma (n = 14) tissues, tumor tissues (n = 29), and adjacent normal tissues (n = 5) from CRC patients, as well as intestinal epithelial tissues from healthy individuals (n = 5) using Circle-Seq technology **(**Fig. 1 and Supplementary Table 1). We found that the abundance of eccDNA gradually increased with the progression of CRC. It was highest in tumor tissues, followed by adenoma, whereas there was no significant difference in eccDNA abundance among intestinal epithelial tissues from healthy individuals, normal tissues from CRC patients, and polyp tissues (Fig. 2A and Supplementary Fig. 1A). The eccDNA abundance in advanced CRC samples are significantly higher than in early CRC samples (Supplementary Fig. 1B). The size distribution of eccDNA in colorectal polyps, adenomas, and tumor tissues was comparable, ranging 300–700 bp (Fig. 2B, Supplementary Figs. 1C, D and 2). The majority of eccDNA fragments were shorter than 1200 bp; however, eccDNA in normal tissues from CRC patients and intestinal epithelial tissues from healthy individuals exhibited significantly different length distributions compared to that in colorectal polyps, adenomas, and tumor tissues, with a major peak of approximately 50 bp (Fig. 2B, Supplementary Figs. 1E and 2). Since there was only a difference in abundance of eccDNA in polyp and adenoma tissues, while there was no significant difference in other eccDNA properties (Fig. 2A, B and Supplementary Fig. 1C), we grouped polyps and adenoma together in the following analysis.

To understand the origins of eccDNA, we aligned the eccDNA sequences to the chromosomes. The alignment revealed a uniform distribution of eccDNA across most autosomes, with notably lower coverage on the Y chromosome (Fig. 2C and Supplementary Fig. 3). This indicates that the DNA sequence on the Y chromosome is more conserved. Except for the Y chromosome, eccDNA frequencies differed significantly across all other chromosomes between the tumor tissues and the precancerous lesion tissues (Fig. 2D). An analysis of the gene‐wide distribution of eccDNA revealed that the tumor tissues exhibited significantly higher levels in the promoter, intron, exon, 5′-UTR, and 3′-UTR regions compared with the precancerous lesion tissues (Fig. 2E). In normal tissues from CRC patients and intestinal epithelial tissues from healthy individuals, eccDNA was primarily derived from the promoter and intron regions, whereas in polyps, adenomas, and tumor tissues, eccDNA was enriched in the promoter and exon regions (Supplementary Fig. 4). The stability of DNA is related to its GC content (Shibata et al. 2012). The GC content of eccDNA in the tumor tissues was significantly higher compared with that in precancerous lesion tissues. Furthermore, the GC content of eccDNA was higher than the immediate upstream or downstream flanking regions (Fig. 2F, G). Overall, the results indicate that eccDNA evolves dynamically during the CRC development.

### Variation of genes carried by eccDNA during colorectal cancer progression

**Fig. 3:**
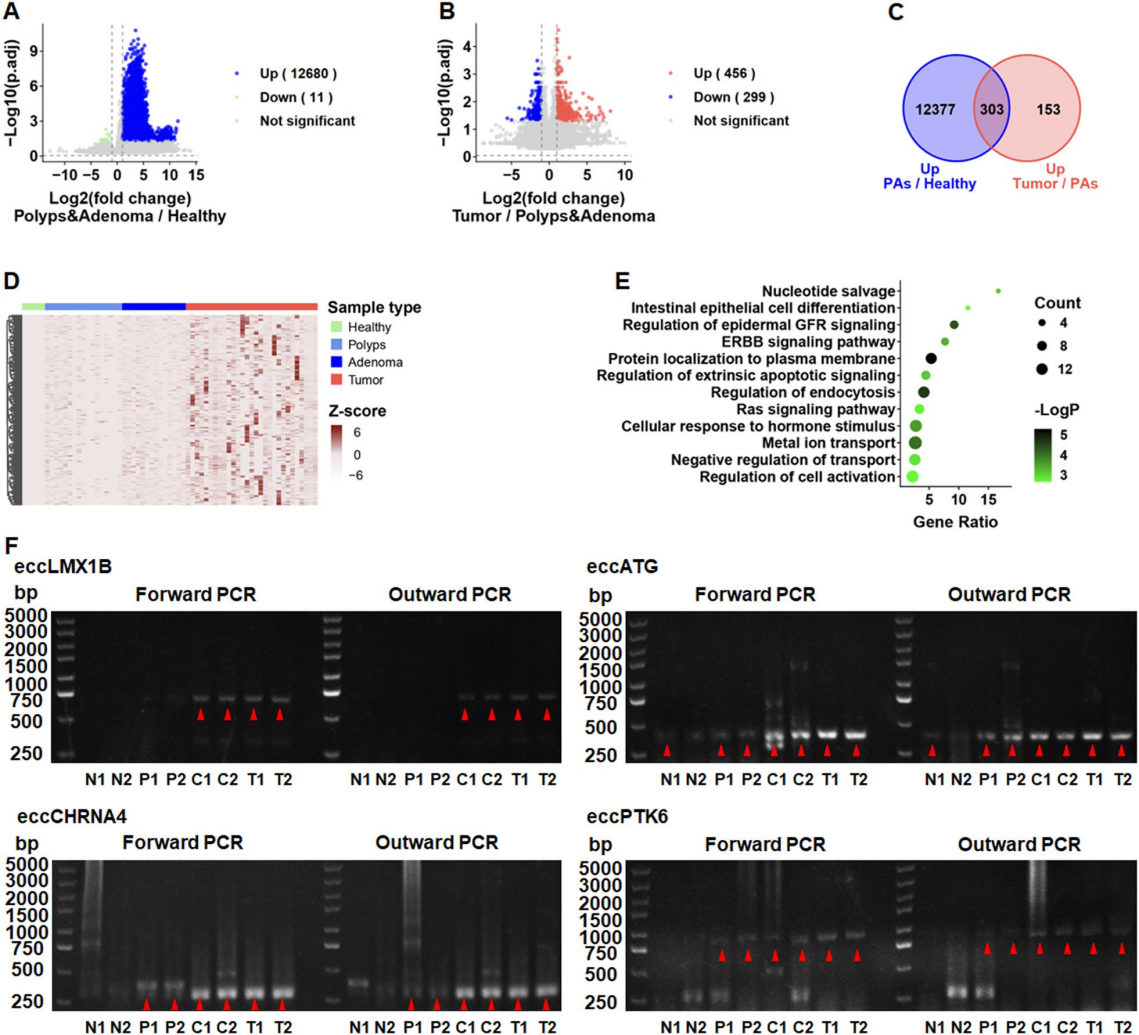
Genomic functional annotation of small eccDNA. **A** Volcanic graph analysis of eccDNA-related genes in colorectal polyp and adenoma tissues (Polyps & Adenoma) and intestinal epithelial tissues (Healthy) analyzed by Circle-seq. **B** Volcanic graph analysis of eccDNA-related genes in tumor tissues (Tumor) and colorectal polyp and adenoma tissues (Polyps & Adenoma). **C** Intersection of eccDNA-related genes upregulated in PAs tissues (polyp and adenoma tissues) compared to healthy tissues (intestinal epithelial tissues) and eccDNA upregulated in tumor tissues compared to PAs tissues (polyp and adenoma tissues). **D** Heatmap showing the 303 eccDNAs that were upregulated throughout CRC progression. **E** Gene Ontology (GO) and Kyoto Encyclopedia of Genes and Genomes (KEGG) pathway analysis of 303 eccDNAs that were upregulated throughout CRC progression. **F** Representative gel images for four highly abundant genes (eccLMX1B, eccCHRNA4, eccATG, eccPTK6) among the 303 eccDNA-related genes validated by outward PCR. N, adjacent normal tissues; P, polyp tissues; C, adenoma tissues; T, tumor tissues

To examine the various genes carried by eccDNA during CRC progression, we performed a differential analysis of Circle-Seq data. A total of 12,680 eccDNAs in the precancerous tissues were upregulated compared with the intestinal epithelial tissues from healthy individuals (Fig. 3A), and 456 eccDNAs in the tumor tissues were upregulated compared with that in the precancerous tissues (Fig. 3B). Of these, 303 eccDNA-related genes were upregulated throughout the progression of CRC (Fig. 3C, D). KEGG pathways showed that the eccDNA-related genes were more likely to be related with the development and metastasis of tumors and classical signaling pathways, such as the nucleotide salvage pathway, intestinal epithelial cell differentiation pathway, regulation of the epidermal GFR signaling pathway, and ERBB signaling pathway (Fig. 3E). To validate the candidate tissues, we selected four highly abundant eccDNA-related genes (*eccLMX1B*, *eccCHRNA4*, *eccATG*, and *eccPTK6*) and verified their circular structures by PCR with divergent primers and the forward primer as a control. The primers were designed according to the breakpoint sequence (Fig. 3F and Supplementary Fig. 5). The results indicated that these eccDNAs increased gradually over the various stages of CRC progression, consistent with the findings from Circle-Seq analysis.

### Oncogene expression is influenced by partial eccDNA-mediated transcriptional regulation

**Fig. 4:**
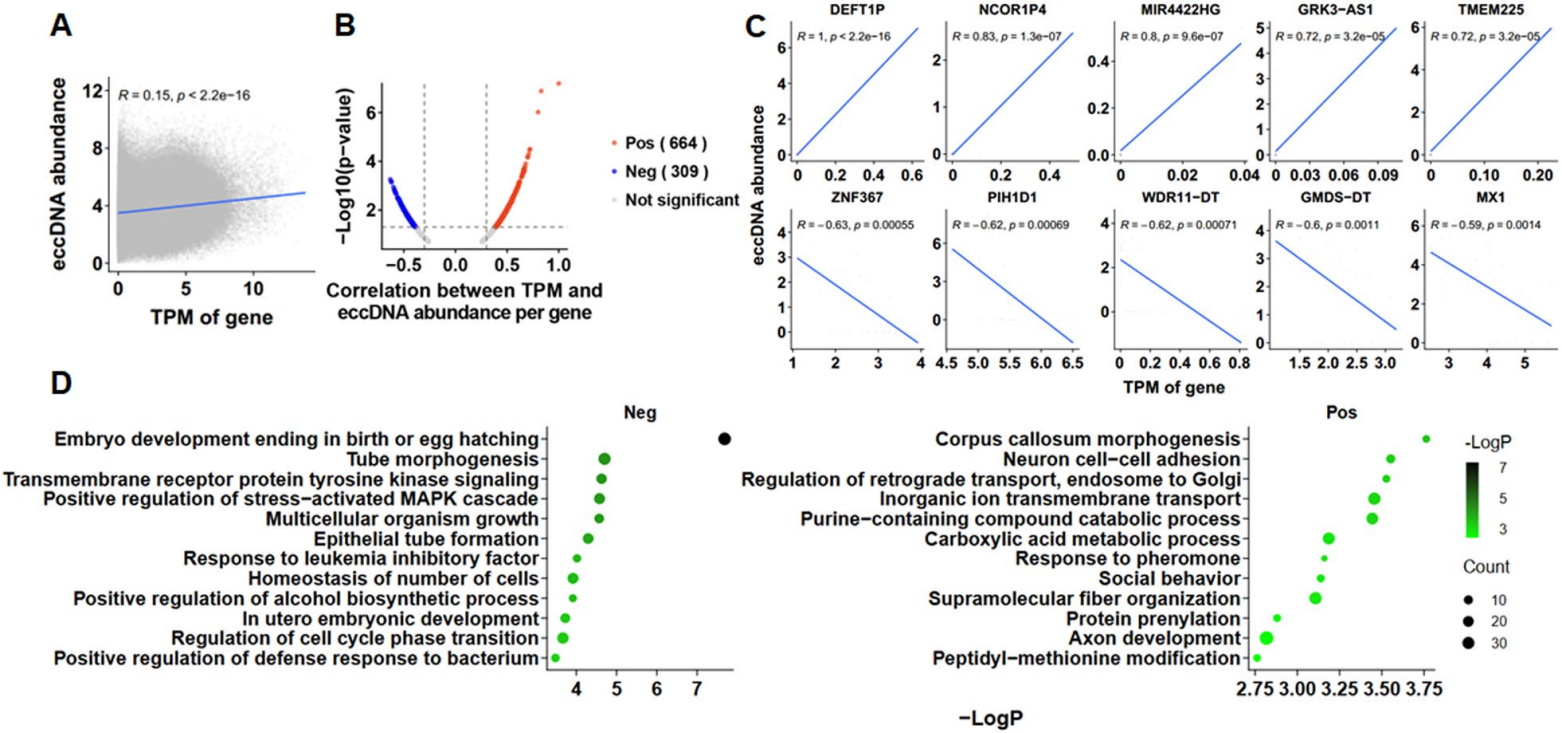
Joint analysis of Circle-seq and RNA-seq. **A** Correlation between the transcription level of the gene carried by the eccDNA and the abundance of the corresponding eccDNA. **B** Correlation between TPM and eccDNA abundance per gene. **C** Correlation of eccDNA abundance with transcription levels of the 10 most significant genes. **D** Gene Ontology (GO) and Kyoto Encyclopedia of Genes and Genomes (KEGG) pathway analysis of the eccDNA-related genes that were negatively (left) or positively (right) correlated with gene expression. TPM, the counts per length of transcript (kb) per million reads mapped

To determine the correlation between eccDNA and gene expression, we performed simultaneous RNA sequencing of tumor tissue samples and found that the overall abundance of eccDNA was positively correlated with gene expression (Fig. 4A). The abundance of 664 eccDNAs was positively correlated with RNA transcription, such as *DEFT1P*, *NCOR1P4*, *MIR4422HG*, GRK3-AS1 and *TMEM225*, whereas 309 eccDNAs were negatively correlated, including *ZNF367*, *PIH1D1*, *WDR11-DT*, GMDS-DT and *MX1* (Fig. 4B, C). EccDNA-related genes were negatively correlated with gene expression and were considerably enriched in embryo development ending in birth or egg hatching, tube morphogenesis, transmembrane receptor protein tyrosine kinase signaling, and positive regulation of the stress-activated MAPK cascade (Fig. 4D, left). eccDNA-related genes, which were positively correlated with gene expression, were considerably enriched in the pathway of corpus callosum morphogenesis, neuron cell–cell adhesion, regulation of retrograde transport, and inorganic ion transmembrane transport (Fig. 4D, right).

### Repetitive sequences contribute to the formation of eccDNA

**Fig. 5:**
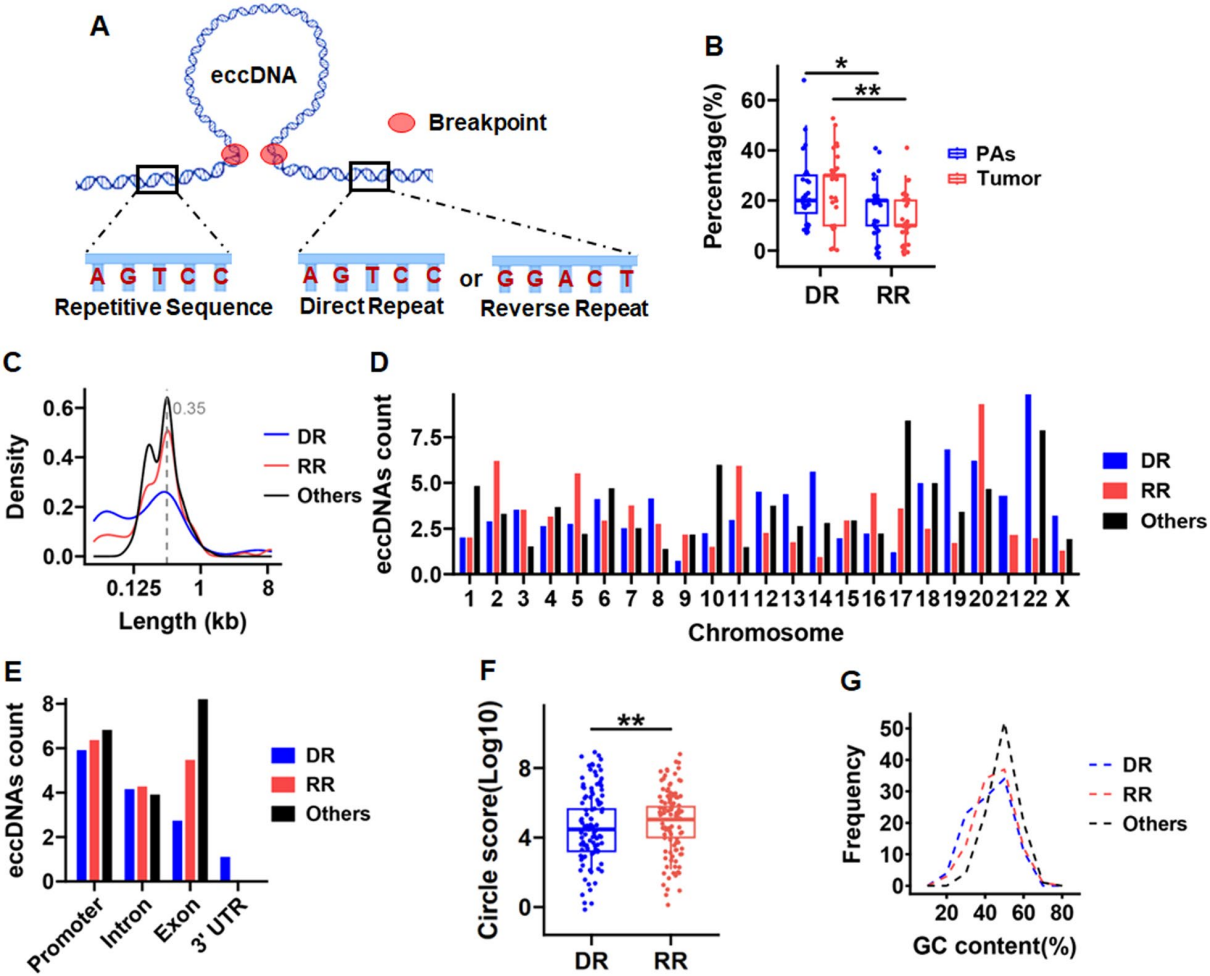
Presence of repetitive sequences near the termini breakpoint of eccDNA. **A** Schematic diagram of direct and reverse repeats in the genesis of eccDNA. **B** Conducts a proportional analysis contrasting direct reverse inverted repeats within eccDNA extracted from colorectal polyp and adenoma tissues (PAs) and tumor tissues (Tumor). **C** Density distributions of direct repeat eccDNA (DR), reverse repeat eccDNA (RR) and eccDNA lacking direct repeats (others). **D** Overall chromosomal distribution of direct repeat eccDNA (DR), reverse repeat eccDNA (RR) and eccDNA lacking direct repeats (others) across the genome. **E** Genomic elements distribution of direct repeat eccDNA (DR), reverse repeat eccDNA (RR) and eccDNA lacking direct repeats (others). **F** Circle Score quality of direct repeat eccDNA (DR) and reverse repeat eccDNA (RR). **G** GC content distribution of direct repeat eccDNA (DR) and reverse repeat eccDNA (RR). Data were statistically analyzed using a Student t test. *p < 0.05, **p < 0.01

To determine the mechanism of eccDNA biogenesis, we focused on the sequence upstream and downstream of the eccDNA termini breakpoint, expanding both ends outward by 100 bp and inward by 100 bp, which may be related to the formation of eccDNA (Shibata et al. 2012; Zhuang et al. 2024). The presence of direct and reverse repeats was detected in eccDNA from both precancerous and tumor tissues, with the frequency of direct repeats higher than reverse repeats (Fig. 5A, B and Supplementary Fig. 6). We next analyzed the differences between direct repeat eccDNA (DR-eccDNA), reverse repeat eccDNA (RR-eccDNA), and eccDNA lacking direct repeats (other-eccDNA). The results showed consistency in terms of size (Fig. 5C). To gain insight into the chromosomal origins of these three types of eccDNA, we aligned their sequences with the chromosomes. We found that DR-eccDNA exhibited higher coverage on chromosomes 14, 19, and 22. RR-eccDNA had higher coverage on chromosomes 2, 11, and 20, whereas the other-eccDNA showed higher coverage on chromosomes 10, 17, and 22 (Fig. 5D). We examined the distribution of these three types of eccDNA in the human genome, which revealed that DR-eccDNA and RR-eccDNA were enriched in the promoter region, whereas other-eccDNA were enriched in the exon region (Fig. 5E). Interestingly, RR-eccDNA showed a significantly higher circle score (Fig. 5F) and GC content (Fig. 5G) compared with DR-eccDNA, suggesting that the circular structure of RR-eccDNA was more stable and intact. This may contribute to the biological function of eccDNA. Overall, examining the role of repeats in eccDNA generation contributes to elucidating the underlying mechanism of eccDNA formation and provides clues for studying the function of eccDNA under physiological and pathological conditions.

### The potential of eccDNA for the diagnosis of colorectal cancer

**Fig. 6:**
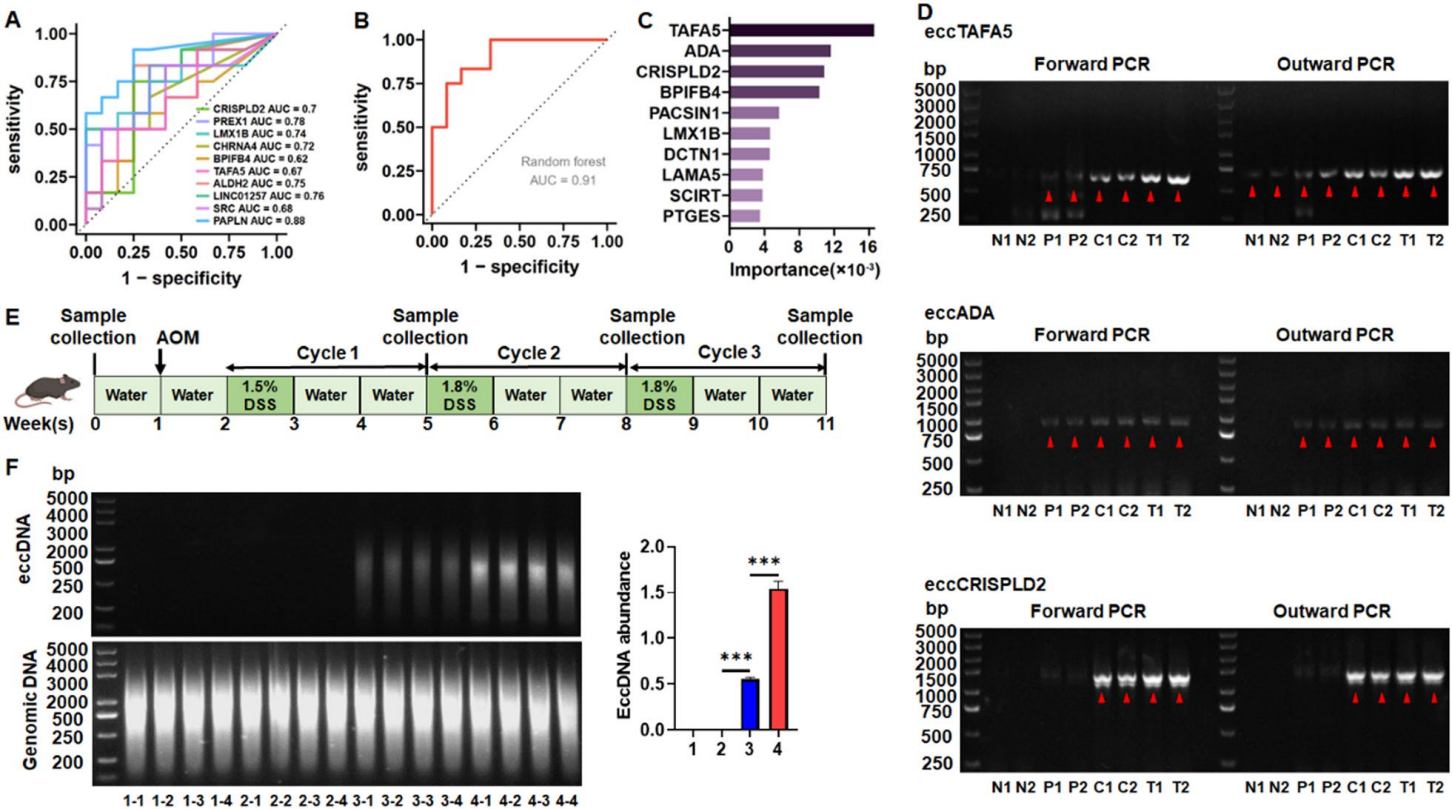
The diagnostic value of eccDNA for CRC. **A** Receiver operating characteristic (ROC) curve of generalized linear model in which individual genes were used as predictors, the abscissa is the false positive rate (1-specificity), the ordinate is the true positive rate (sensitivity), and the AUC (area under curve) is the area enclosed by the coordinate axis under the ROC curve. **B** ROC curves of random forest model predicted by multiple gene combinations. **C** The 10 most important characteristic genes in the random forest model predicted by multi-gene combinations, the abscissa represents the importance score. **D** Representative gel images for validated eccDNA (eccTAFA5, eccADA, eccCRISPLD2) originating from the three most important characteristic genes in the random forest model were predicted by multi-gene combinations by outward PCR. N, adjacent normal tissues; P, polyp tissues; C, adenoma tissues; T, tumor tissues. **E** Schematic of the experimental strategy for AOM/DSS-induced carcinogenesis. **F** EccDNA abundance after removal of linear DNA detected by agarose gel electrophoresis in different stages in the progression of CRC. 1, Samples collected prior to AOM/DSS treatment (Week 0); 2, Samples collected after the end of the first cycle of AOM/DSS treatment (Week 5); 3, Samples collected after the end of the second cycle of AOM/DSS treatment (Week 8); 4, Samples collected after the end of the third cycle of AOM/DSS treatment (Week 11). Data were statistically analyzed using a Student t test. ***p < 0.001

To determine the ability of eccDNA-related genes to distinguish between polyps, adenoma, and tumor tissues, we selected 10 genes with the highest eccDNA abundance from previous 303 genes that were continuously upregulated during tumor progression. The AUC of the generalized linear model using individual genes as predictors was mostly < 0.8, suggesting the potential of combining these genes for prediction (Fig. 6A). Compared with the generalized linear model, the random forest model showed a stronger generalization ability and could evaluate the importance of each feature to prediction. Therefore, random forest was used to construct a multi-gene combination prediction model. The results indicated that the AUC of the random forest model reached 0.91, which had a better ability to distinguish between precancerous lesions (polyps and adenomas) and tumors (Fig. 6B). We also demonstrated 10 genes, TAFA5, ADA, CRISPLD2, BPIFB4, PACSIN1, LMX1B, DCTN1, LAMA5, SCIRT, PTGES(Fig. 6C and Supplementary Fig. 7) that are most important for the classification of the model and verified the circular structure by outward PCR (Fig. 6D and Supplementary Fig. 8). These gene-specific eccDNAs have potential to be used as tumor biomarkers.

To further validate our findings, we established a primary colorectal cancer model induced by azoxymethane/dextran sodium sulfate (AOM/DSS) treatment using C57BL/6 mice (Fig. 6E). The eccDNA was collected from mouse intestinal tissues at weeks 0, 5, 8, and 11, and gel electrophoresis was performed following amplification to identify the changes in eccDNA during CRC development. Consistent with the results of Circle-Seq sequencing, the eccDNA abundance gradually increased with the extension of tumor induction time during the process of tumor formation (Fig. 6F). The results indicate the potential of gene-specific eccDNA as a biomarker for the diagnosis of CRC.

## Discussion

EccDNA is ubiquitous in normal tissues, tumor tissues, and body fluids of the human body (Møller et al. 2018; Paulsen et al. 2018). Although the abundance of eccDNA in normal tissues is relatively lower compared with tumor tissues, normal tissues typically contain small eccDNA ranging from a few dozen base pairs to several thousand bases (Sin et al. 2020; Luo et al. 2023; Paulsen et al. 2018). In this study, we identified eccDNA in normal intestinal epithelial tissue, polyps, adenomas, and tumor tissues using Circel-seq. Notably, eccDNA is closely related to the progression of CRC, and there are differences in the various stages of CRC occurrence and development. The abundance of eccDNA increased with the progression of tumors. In addition, eccDNA in the polyps, adenomas, and tumor tissues exhibited a larger circular size compared with normal intestinal epithelial tissue. We found that eccDNA in polyp and adenoma tissues is similar except for abundance, which may be related to their biological properties, molecular mechanisms, and continuity of disease progression. Polyps and adenomas, both precancerous lesions of colorectal cancer, have similar cellular origins, and they may share certain characteristics at the molecular level, including the mechanism of eccDNA formation. In addition, polyps are a precursor to adenomas, the characteristics of eccDNA may not have changed significantly during this disease progression. However, there were similarities in other eccDNA features, such as GC content and chromosomal origin. We also observed that genes, such as *TAFA5*, *ADA*, and *CRISPLD2*, produce eccDNA throughout the occurrence and development of CRC. Because of the unique structure of eccDNA, it may be free from some regulatory mechanisms on chromosomes. In normal cells, the expression of oncogenes is tightly regulated; however, in tumor cells, the eccDNA-related genes are amplified in large quantities. This amplification may regulate the expression of numerous downstream genes, promote cell proliferation, and inhibit cell differentiation, which results in tumor progression. Taken together, these findings reveal the dynamic changes of eccDNA during tumor progression, although the biological function of eccDNA must be further elucidated.

Our research also focused on repetitive sequences near the termini breakpoint of eccDNA. We found that most eccDNA contained direct and reverse repeats near the termini breakpoint, which is consistent with previous studies (Luo et al. 2023; Shibata et al. 2012; Zhuang et al. 2024). Repetitive sequences contribute to the formation of eccDNA. Their mechanisms are diverse and involve multiple biological processes, such as homologous recombination, non-homologous end joining, microhomologous-mediated end joining, and mismatch repair (Yang et al. 2022; Rose, et al. 2024). A large fraction of the eccDNA molecule still lacks repetitive sequences, suggesting the involvement of DNA damage repair pathways in eccDNA formation. In addition, differences in eccDNA signatures in tumor tissues, precancerous tissues, and normal intestinal epithelial tissues suggest that the mechanisms of eccDNA formation vary at different stages of CRC progression. The variation may be the result of the accumulation of genetic mutations and increased genomic instability during progression from polyps to CRC. The presence and specific arrangement of repetitive sequences provide a structural and mechanistic basis for the formation of eccDNA, which is of great significance in understanding the role of eccDNA in physiological and pathological conditions.

We found the presence of eccDNA derived from genes, such as *TAFA5*, *ADA*, and *CRISPLD2*, throughout the occurrence and development of CRC, with the only difference being the eccDNA abundance. Therefore, these gene-specific eccDNAs may serve as biomarkers for the early diagnosis of CRC. Compared with traditional tumor markers, such as protein markers, circulating tumor cells (CTC), circulating tumor DNA (ctDNA), and extracellular vesicles (EV), eccDNA has significant advantages in terms of specificity and revealing tumor heterogeneity, particularly in real-time monitoring (Ignatiadis et al. 2021; Alix-Panabières and Pantel 2021; Kalluri and McAndrews 2023). EccDNA may increase the efficiency of cancer diagnosis and improve patient outcomes. In addition, eccDNA carries tumor-specific genetic variant information, which may reveal the genomic characteristics of the tumor and guide personalized treatment strategies. Although eccDNA has great potential as a diagnostic marker for tumors, there are limitations to its practical application, such as the immaturity of eccDNA detection and analysis technology, the lack of standardization, high cost, and technical requirements. Before eccDNA can become a reliable clinical tool, there are a series of technical, standard, and application challenges that require optimization and technological innovation to overcome. Furthermore, this study is limited due to its relatively small sample size. Expanding the sample size to validate our findings and identifying more tumor-specific eccDNA is the focus of our future work.

In conclusion, we conducted a comprehensive analysis of the characteristics of eccDNA during CRC progression. We showed dynamic behavior of eccDNA at different stages of CRC occurrence and development: polyps, adenomas, and cancers. This emphasizes the importance of eccDNA in the process of CRC progression and provides a theoretical basis for the development of CRC-specific eccDNA biomarkers.

## Supplementary Information

Additional file 1.

## Data Availability

The data presented in this study are available upon request from the corresponding author.
