# Supplementary material for: Extrachromosomal circular DNA as a novel biomarker for the progression of colorectal cancer
Source: Mol Med. 2025 Apr 1;31:123. doi: 10.1186/s10020-025-01164-y (PMC11960012; doi:10.1186/s10020-025-01164-y)
Supplement: Supplementary file 1 — Additional file 1. [file 10020_2025_1164_MOESM1_ESM.docx]

| 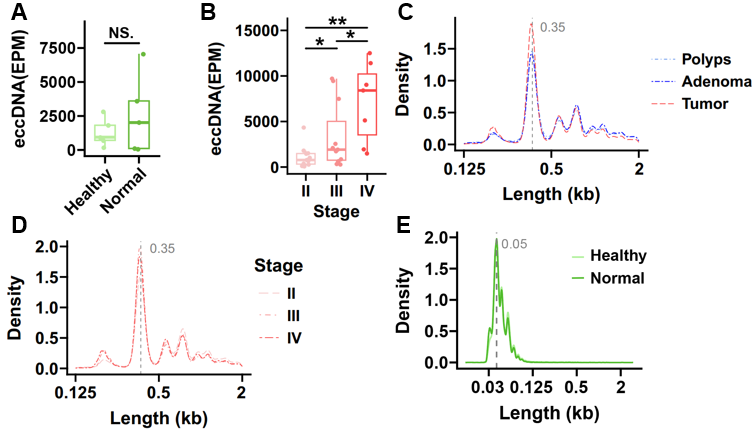 |
| --- |
| Supplementary Figure 1. The general characteristics of eccDNA. (A) EccDNA abundance after removal of linear DNA in normal tissues (Normal) from CRC patients and intestinal epithelial tissues from healthy individuals (Healthy). (B) EccDNA abundance after removal of linear DNA in different stages of CRC. (C) Density distributions of eccDNA in polys, adenoma and tumor samples. (D) Density distributions of eccDNA in different stages of CRC. (E) Density distributions of eccDNA in normal tissues (Normal) from CRC patients and intestinal epithelial tissues from healthy individuals (Healthy). Data were statistically analyzed using a Student t test. *p < 0.05, **p < 0.01, ***p < 0.001. |

| 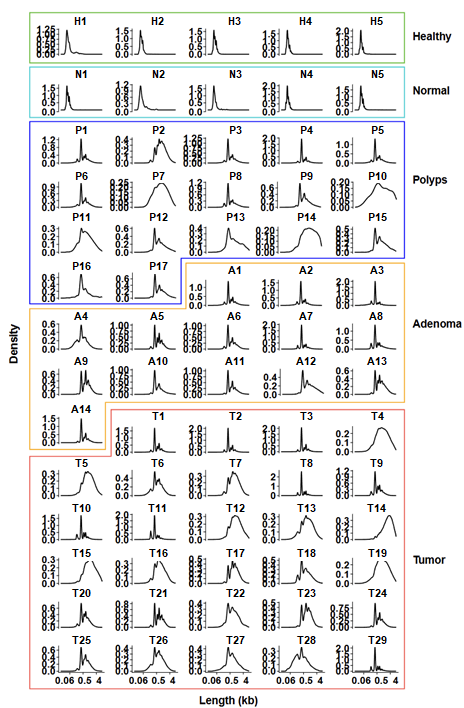 |
| --- |
| Supplementary Figure 2. Density distributions of eccDNA in each clinical sample. |

| 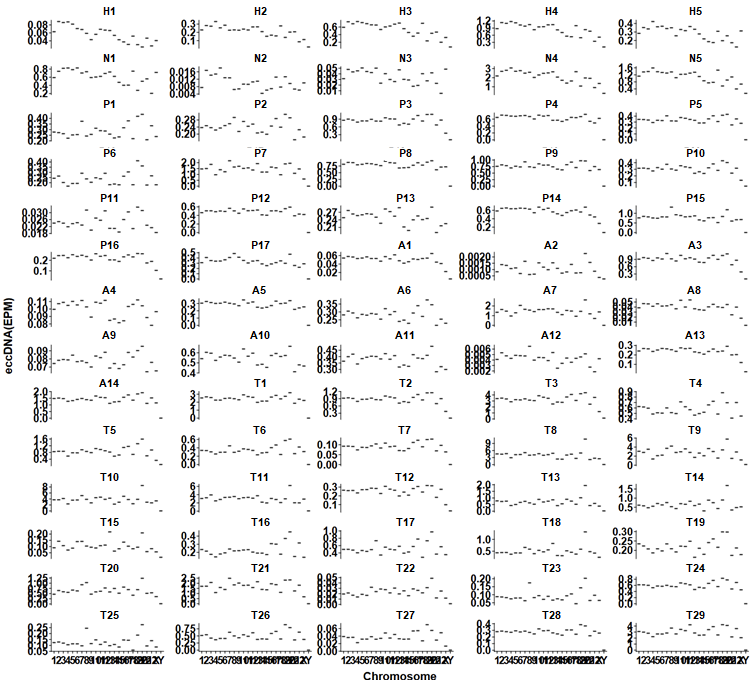 |
| --- |
| Supplementary Figure 3. Differences in chromosomal origin of eccDNA in each clinical sample. |

| 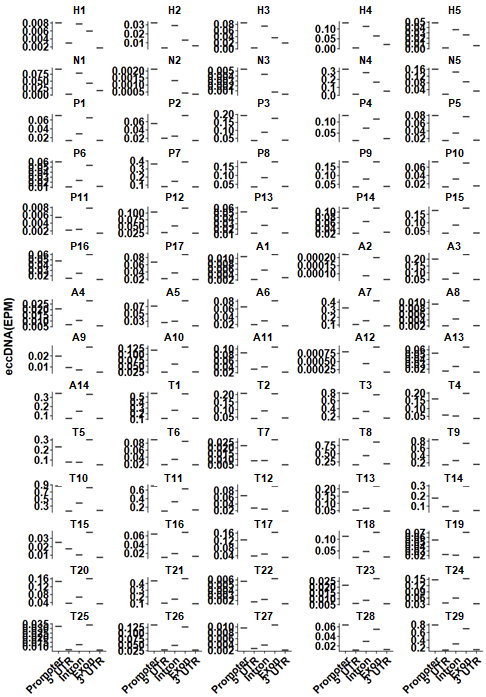 |
| --- |
| Supplementary Figure 4. Genomic elements distribution of eccDNA in each clinical sample. |

| 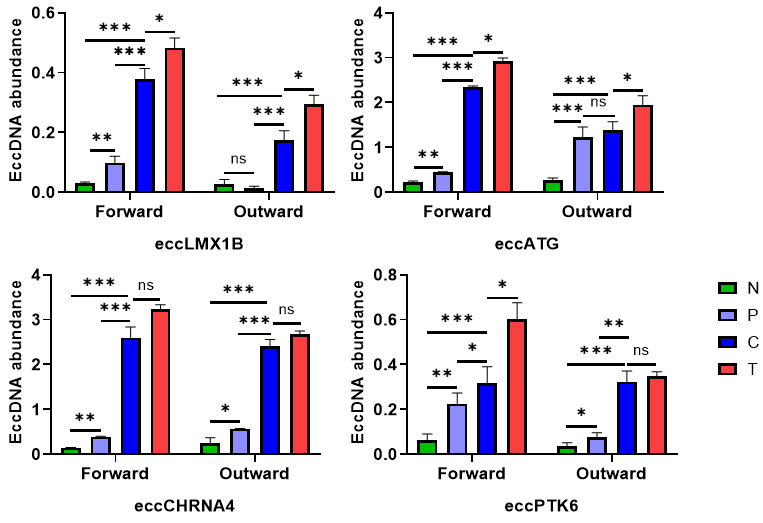 |
| --- |
| Supplementary Figure 5. Validation of eccLMX1B, eccCHRNA4, eccATG, eccPTK6. Quantification of gel images for four highly abundant genes (eccLMX1B, eccCHRNA4, eccATG, eccPTK6) among the 303 eccDNA-related genes validated by outward PCR. N, adjacent normal tissues; P, polyp tissues; C, adenoma tissues; T, tumor tissues. |

T1_chr2_95057339_95057980

TGGCCTGTGACTTCCCAGGCTGTGGCCCCCTTGCTTCAGGGGGCGATCCTGGGAGTATCCTCCTGGTGGCCTTCCTTGTGCAGGGCAGGGTGGCCTGGGA-eccDNA-CGGGGGTTCCTAGCTGTGGCTGCTCTCTTCCTTCACAGGTAACGAGATCATCACACTGCCAGGCTCTCTAGAGGGTACCAGAGAGGTTGGATTCACACGG

T5_chr18_75086495_75086810

TGAATCCATGTGTGAGCATGTGCATGTGAATGTACATATGTGAGCATGAGTGTGTTCATATTTACACATGTGCTGTGCATGCCTGTGTGAATGTGCATGC-eccDNA-ACATGTACATGTGTGCATCTGTGTACATGTGAATGTGTGCATGCATGTGTGTGACTTCACATGTGAGGGCATGTGTGTGAATATGCATGTGTGTGGGCAT

T6_chr6_145212329_145212647

CTCCTGGGTTCAAGCAATTCTCCTGCCTCAGCCTCCCGAGTAGCTGGGACTATAGGTGCGCGCCACCATGCCCAGCTAATTTTTGTAGAGATTTAGTAGA-eccDNA-CTACTAAAAAATACAAAAATTGGCTGGGTGTGGTGGCATGCGCTTGTAGTCCCAGCTACTCAGGAGGCTGAGGCAGGAAAATTGCTTGAACCCAGGAGGT

A5_chr7_93895329_93895503

AGTGAATGATGAGAATTAATTGAGCCCAGTGATTTGGGGATACATATTATGTAACATAGGTATTTAAGGAGTTACACTGATAACTCCTTAATAAACAAAG-eccDNA-TTACACATCTGCTAAGAAAGATAGTTAATCTCTTCTGAGCAGTAAATGGTCATGAGGGTTTTGCTAAAGTTTAAGCAACTCCTTCACCAACTAGGTATTT

A13_chr20_166536_166897

TAGTAAAGATATTAGATTTTAATATAACACATCAATAATTATATTTAATATAAATGATCTAAATCAGGGGTCCCCACCCCCCAGGCCATGGACCGGTATA-eccDNA-ATTAAACCGGTCCCTGGTGCCAAAAAGGCTGGGGACCACTGATCTAAATACACCAATAAAAAGAAAAAGATTGTAAGATTGGATTTTAAAAGACCTGACT

A14_chr1_35579287_35579556

ATACAAAAATTAGCTGGGTGGGGTGGCAGGCACCTGTAATCCCAGCTACTCCGGAGGCTGAGGCAGGAGAATCCCTTGAACCTGCGGGGCGGAGGTTGCA-eccDNA-ACCTCAGCCTCCCAAGTAGCTGGGATTACAGGTACCTGTCACCACGCCCAGCTAATTTTTGTATTTTTAGTAGGGGCGTGGTTTCACCATGTTGGCCAGG

P4_chr2_17691170_17691430

ATAAGAAGATCTTCAAGATATATCACTAAACCAAAACAGCAACTATTCACAATAGCCAAGATATGTAATCAACCCACATGTCCATTAATGAATGAAGAGA-eccDNA-TTTCTTCATTCATTAATGGACATGTGGGTTGATTACATATCTTGGCTATTGTGAATAGTTGCCGTTTTGAACCAACGACAAAAAACACATGACTCTCAAT

P7_chr2_17691160_17691432

ATGTACCAACATAAGAAGATCTTCAAGATATATCACTAAACCAAAACAGCAACTATTCACAATAGCCAAGATATGTAATCAACCCACATGTCCATTAATG-eccDNA-TCTTCATTCATTAATGGACATGTGGGTTGATTACATATCTTGGCTATTGTGAATAGTTGCCGTTTTGAACCAACGACAAAAAACACATGACTCTCAATAG

P9_chr15_64715314_64715674

CAGTGGCGTGATCTCGGCTCACTGCAAGCTCCGCCTCCCAGGTTCACGCCATTCTCCTGCCTCAGCCTCCTGAGTAGCTGGGACTACAAGTGCCCGCCAC-eccDNA-TGGCCAACATGGTGAAATCCTGAGTCCACCAAAAATTAGCCAGGCGTGATGGCATGTACCTGTAGTCCCAGCTACTTGAGAGGCTGAGGTGGGAAAATCA

P12_chr3_110723170_110724108

TGTTGCAATCTGTCTCAACACTAATATAGGATGTAATATATCATTATACATTTAAGATATAATATGTCATTACATCTTAAAAATAATAATTACAATAATC-eccDNA-ATTTCAAAATGATCTTTTGTTTACTATATTGAATAATATTATTAAGATATGTTCCTGAACAGAGTTTTAATTTAAATATTAAGCAAAAAGAAAGGGTCTC

Supplementary Figure 6. Representative repetitive sequences. Red, direct repeat; Green, reverse repeat.

| 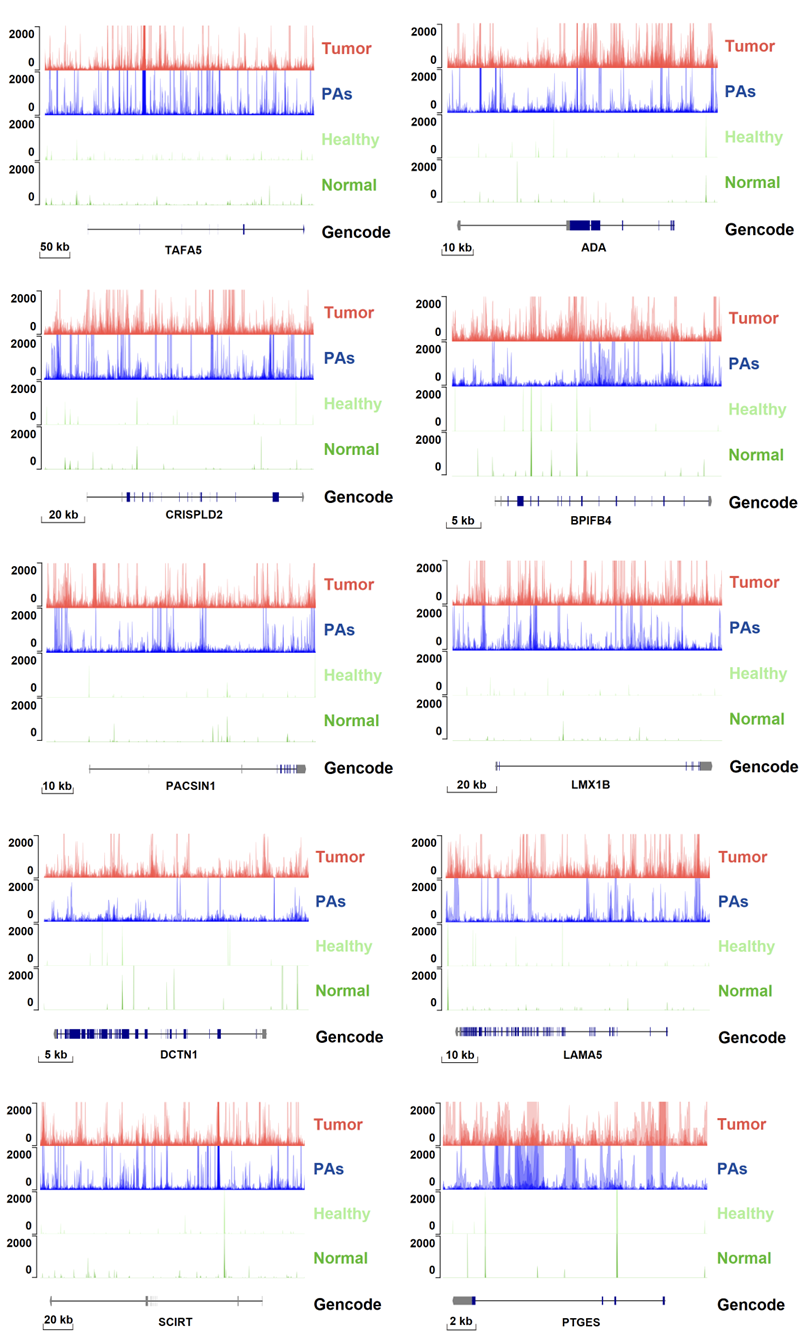 |
| --- |
| Supplementary Figure 7. Integrative Genomics Viewer (IGV) depicted for eccDNAs derived from the 10 most important characteristic genes in the random forest model predicted by multi-gene combinations |

| 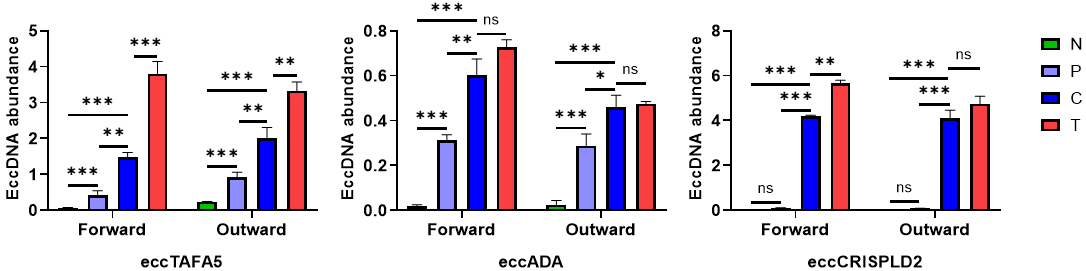 |
| --- |
| Supplementary Figure 8. Validation of eccTAFA5, eccADA, eccCRISPLD2. Quantification of gel images for validated eccDNA (eccTAFA5, eccADA, eccCRISPLD2) originating from the three most important characteristic genes in the random forest model were predicted by multi-gene combinations by outward PCR. N, adjacent normal tissues; P, polyp tissues; C, adenoma tissues; T, tumor tissues. |

| **Supplementary Table 1. Basic clinical information of samples for Circle-seq.** | | | | | |  |  |  |  |  |  |
| --- | --- | --- | --- | --- | --- | --- | --- | --- | --- | --- | --- |
| Diagnosis | Sample number | Sample Type | Gender | Age | BMI | Stage |  |  |  |  |  |
| colorectal cancer | T1 | tumor tissues | male | 53 | 22.8 | II |  |  |  |  |  |
|  | N1 | adjacent normal tissues |  |  |  |  |  |  |  |  |  |
| colorectal cancer | T2 | tumor tissues | male | 34 | 19.9 | IV |  |  |  |  |  |
|  | N2 | adjacent normal tissues |  |  |  |  |  |  |  |  |  |
| colorectal cancer | T3 | tumor tissues | male | 54 | 21.7 | IV |  |  |  |  |  |
|  | N3 | adjacent normal tissues |  |  |  |  |  |  |  |  |  |
| colorectal cancer | T4 | tumor tissues | male | 40 | 21.5 | II |  |  |  |  |  |
|  | N4 | adjacent normal tissues |  |  |  |  |  |  |  |  |  |
| colorectal cancer | T5 | tumor tissues | male | 60 | 21.9 | IV |  |  |  |  |  |
|  | N5 | adjacent normal tissues |  |  |  |  |  |  |  |  |  |
| colorectal cancer | T6 | tumor tissues | male | 45 | 21.3 | II |  |  |  |  |  |
| colorectal cancer | T7 | tumor tissues | male | 83 | 21.8 | IV |  |  |  |  |  |
| colorectal cancer | T8 | tumor tissues | female | 70 | 19.6 | II |  |  |  |  |  |
| colorectal cancer | T9 | tumor tissues | male | 85 | 22.1 | II |  |  |  |  |  |
| colorectal cancer | T10 | tumor tissues | female | 64 | 22.9 | II |  |  |  |  |  |
| colorectal cancer | T11 | tumor tissues | female | 50 | 25.9 | II |  |  |  |  |  |
| colorectal cancer | T12 | tumor tissues | male | 69 | 27.9 | III |  |  |  |  |  |
| colorectal cancer | T13 | tumor tissues | male | 59 | 22.3 | III |  |  |  |  |  |
| colorectal cancer | T14 | tumor tissues | male | 51 | 21.5 | III |  |  |  |  |  |
| colorectal cancer | T15 | tumor tissues | male | 62 | 22.89 | III |  |  |  |  |  |
| colorectal cancer | T16 | tumor tissues | female | 55 | 29 | IV |  |  |  |  |  |
| colorectal cancer | T17 | tumor tissues | male | 67 | 20.8 | III |  |  |  |  |  |
| colorectal cancer | T18 | tumor tissues | female | 69 | 26.5 | IV |  |  |  |  |  |
| colorectal cancer | T19 | tumor tissues | male | 53 | 26 | IV |  |  |  |  |  |
| colorectal cancer | T20 | tumor tissues | female | 66 | 21.8 | II |  |  |  |  |  |
| colorectal cancer | T21 | tumor tissues | male | 55 | 24.2 | III |  |  |  |  |  |
| colorectal cancer | T22 | tumor tissues | female | 58 | 24.6 | III |  |  |  |  |  |
| colorectal cancer | T23 | tumor tissues | female | 55 | 25.9 | II |  |  |  |  |  |
| colorectal cancer | T24 | tumor tissues | female | 66 | 20.8 | III |  |  |  |  |  |
| colorectal cancer | T25 | tumor tissues | male | 84 | 30.3 | III |  |  |  |  |  |
| colorectal cancer | T26 | tumor tissues | female | 50 | 20 | III |  |  |  |  |  |
| colorectal cancer | T27 | tumor tissues | male | 73 | 29.3 | III |  |  |  |  |  |
| colorectal cancer | T28 | tumor tissues | male | 63 | 20.7 | II |  |  |  |  |  |
| colorectal cancer | T29 | tumor tissues | male | 74 | 19 | II |  |  |  |  |  |
| colorectal adenoma | A1 | adenoma tissues | female | 55 | 20.4 | - |  |  |  |  |  |
| colorectal adenoma | A2 | adenoma tissues | male | 43 | 29 | - |  |  |  |  |  |
| colorectal adenoma | A3 | adenoma tissues | female | 51 | 27.7 | - |  |  |  |  |  |
| colorectal adenoma | A4 | adenoma tissues | female | 57 | 22.8 | - |  |  |  |  |  |
| colorectal adenoma | A5 | adenoma tissues | female | 47 | 25.9 | - |  |  |  |  |  |
| colorectal adenoma | A6 | adenoma tissues | female | 57 | 24.2 | - |  |  |  |  |  |
| colorectal adenoma | A7 | adenoma tissues | female | 60 | 19.9 | - |  |  |  |  |  |
| colorectal adenoma | A8 | adenoma tissues | male | 56 | 24.6 | - |  |  |  |  |  |
| colorectal adenoma | A9 | adenoma tissues | female | 58 | 23.5 | - |  |  |  |  |  |
| colorectal adenoma | A10 | adenoma tissues | female | 63 | 21.6 | - |  |  |  |  |  |
| colorectal adenoma | A11 | adenoma tissues | male | 51 | 24.6 | - |  |  |  |  |  |
| colorectal adenoma | A12 | adenoma tissues | male | 50 | 21.4 | - |  |  |  |  |  |
| colorectal adenoma | A13 | adenoma tissues | male | 57 | 19.1 | - |  |  |  |  |  |
| colorectal adenoma | A14 | adenoma tissues | male | 48 | 20.2 | - |  |  |  |  |  |
| colorectal adenoma | A15 | adenoma tissues | female | 52 | 18.9 | - |  |  |  |  |  |
| colorectal adenoma | A16 | adenoma tissues | male | 47 | 21.3 | - |  |  |  |  |  |
| colorectal adenoma | A17 | adenoma tissues | female | 58 | 22.1 | - |  |  |  |  |  |
| colorectal polyp | P1 | polyp tissues | male | 55 | 27.1 | - |  |  |  |  |  |
| colorectal polyp | P2 | polyp tissues | male | 46 | 22.6 | - |  |  |  |  |  |
| colorectal polyp | P3 | polyp tissues | female | 53 | 22.8 | - |  |  |  |  |  |
| colorectal polyp | P4 | polyp tissues | male | 38 | 26 | - |  |  |  |  |  |
| colorectal polyp | P5 | polyp tissues | female | 51 | 23 | - |  |  |  |  |  |
| colorectal polyp | P6 | polyp tissues | male | 46 | 28.8 | - |  |  |  |  |  |
| colorectal polyp | P7 | polyp tissues | male | 40 | 22.6 | - |  |  |  |  |  |
| colorectal polyp | P8 | polyp tissues | female | 51 | 21.2 | - |  |  |  |  |  |
| colorectal polyp | P9 | polyp tissues | female | 39 | 21.8 | - |  |  |  |  |  |
| colorectal polyp | P10 | polyp tissues | male | 49 | 25.7 | - |  |  |  |  |  |
| colorectal polyp | P11 | polyp tissues | female | 45 | 25.9 | - |  |  |  |  |  |
| colorectal polyp | P12 | polyp tissues | male | 47 | 24.7 | - |  |  |  |  |  |
| colorectal polyp | P13 | polyp tissues | male | 55 | 23.8 | - |  |  |  |  |  |
| colorectal polyp | P14 | polyp tissues | female | 42 | 20.3 | - |  |  |  |  |  |
| healthy individual | H1 | intestinal epithelial tissue | male | 43 | 24.5 | - |  |  |  |  |  |
| healthy individual | H2 | intestinal epithelial tissue | male | 51 | 23 | - |  |  |  |  |  |
| healthy individual | H3 | intestinal epithelial tissue | male | 49 | 24.2 | - |  |  |  |  |  |
| healthy individual | H4 | intestinal epithelial tissue | female | 46 | 21.1 | - |  |  |  |  |  |
| healthy individual | H5 | intestinal epithelial tissue | male | 53 | 20.9 | - |  |  |  |  |  |
| Supplementary Table 1. Clinical samples of colorectal polyp (n=17) and adenoma (n=14) tissues, tumor tissues (n=29) and adjacent normal tissues (n=5) from CRC patients and intestinal epithelial tissues from healthy individuals (n=5) for Circle-seq were collected from the above individuals. | | | | | |  |  |  |  |  | - |
|  | | | | | |  |  |  |  |  | - |
